# The impact of low advanced glycation end products diet on obesity and related hormones: a systematic review and meta-analysis

**DOI:** 10.1038/s41598-020-79216-y

**Published:** 2020-12-17

**Authors:** Mohammad Hassan Sohouli, Elham Sharifi-Zahabi, Abolfazl Lari, Somaye Fatahi, Farzad Shidfar

**Affiliations:** 1 Student Research Committee, Faculty of Public Health Branch, Iran University of Medical Sciences, Tehran, Iran; 2 Department of Nutrition, School of Public Health, Iran University of Medical Sciences, Hemmat Superhighway, Tehran, Iran; 3 Pediatric growth and development research center, Iran University of Medical Sciences, Tehran, Iran

**Keywords:** Diseases, Gastroenterology, Nutrition

## Abstract

Several randomized clinical trials (RCTs) have investigated the effect of dietary advanced glycation end products (AGE) on obesity factors and related hormones in adults; results were conflicting. Therefore, a study was performed to assess the effect of low advanced glycation end products diet on obesity and related hormones. A comprehensive literature search without any limitation on language was conducted using the following bibliographical databases: Web of Science, Scopus, Ovid MEDLINE, Cochrane, and Embase up to October, 2019. From the eligible trials, 13 articles were selected for the systematic review and meta-analysis. Our systematic reviews and meta-analyses have shown a significant decrease in BMI (WMD: − 0.3 kg/m^2^; 95% CI: − 0.52, − 0.09, *p* = 0.005; I^2^ = 55.8%), weight (WMD: − 0.83 kg; 95% CI: − 1.55, − 0.10, *p* = 0.026; I^2^ = 67.0%), and leptin (WMD: − 19.85 ng/ml; 95% CI: − 29.88, − 9.82, *p* < 0.001; I^2^ = 81.8%) and an increase in adiponectin (WMD: 5.50 µg/ml; 95% CI: 1.33, 9.67, *p* = 0.010; I^2^ = 90.6%) levels after consumption of the low AGE diets compared to the high AGE diets. Also, the effect of intake of low AGE compared to high AGE diets was more pronounced in subgroup with duration > 8 weeks for the BMI and weight. Overall, according to our results, although low AGE diets appeared to be statistically significant in reducing the prevalence of obesity and chronic diseases compared to high consumption of dietary AGEs. But, no clinical significance was observed. Therefore, to confirm these results clinically, further prospective studies should be conducted in this regard. The study protocol was registered in the in International prospective register of systematic reviews (PROSPERO) database as CRD42020203734.

## Introduction

Overeating and concomitant obesity is a serious public health problem worldwide with close links to chronic health conditions such as diabetes mellitus and cardiovascular diseases^1^. Obesity is associated with Inflammation and oxidative stress, which in turn lead to the genesis of non-communicable chronic diseases^2,3^. Lifestyle modification, with a special focus on dietary pattern, plays an important role in the prevention and control of obesity and its related complication. Recent studies have demonstrated that the consumption of highly processed foods that are extremely high in fat, sugar, salt and potentially toxic compounds known as advanced glycation end-products (AGEs)^4,5^ are associated with increased risk of chronic diseases^6,7^. AGEs are a group of sugar modifications which are formed through the non-enzymatic reaction of sugars with free amino groups of proteins, lipids or nucleic acids^5,8^. AGEs have noticeable pro-inflammatory and prooxidant impacts^9^. Furthermore a positive association between AGEs intake and serum level of AGEs, visceral fat and insulin resistance has been detected, which suggests a casual role of dietary AGEs in metabolic syndrome independent from energy balance^10,11^. When that AGE production binding to receptors AGEs (RAGE), AGEs trigger generation of reactive oxygen species (ROS) and initiate a downstream pro-inflammatory signaling cascade including activation of RAGE/TLR4 (Toll-like receptors 4)-NF-κB-ROS pathways^12^ and contribute to both obesity and related inflammatory diseases^13^. Since diet derived AGEs increase the risk of obesity and related inflammatory diseases, reduced intake of AGEs is thought to be beneficial, independently from the intake of a standard calorie restricted diet^7^. Several studies have shown that consumption of low dietary AGEs is associated with reduced circulating and urinary levels of AGE markers and improved anthropometric, glycemic, cardio metabolic and inflammatory indices in individual with overweight and obese^14–17^. However, the exact mechanism of action of AGEs in obesity-related complications remains unknown. Also there is no established recommendation surrounding the intake of foods with high amount of AGEs such as heat treated cereal or powdered milk which may be considered contributing to a healthy diet. Therefore, this meta-analysis was carried out to analyze the effects of diet-derived AGEs on obesity and related hormones, as well as to discuss the molecular mechanisms of action of these compounds on the development of chronic diseases.

## Results

### Search results

**Figure 1:**
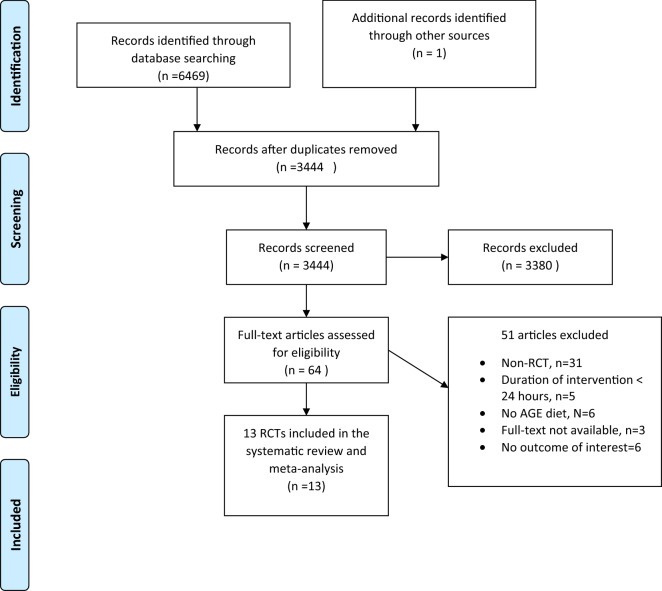
Flow chart of study selection process.

The flow diagram of the process of study selection is shown in Fig. 1. In total, we identified 6469 through database searching and 2 studies identified through other sources. After excluding of duplicates, 3444 papers remained for additional screening based on title and abstract. 3380 one studies were discarded leaving after screening based on title and abstract. Finally, 64 potentially eligible studies for further evaluation that 51 article excluded due to; non-RCT (n = 31), duration of intervention < 24 h (n = 5), no AGE diet (n = 6), full-text not available (n = 3) and no outcome of interest (n = 6). Finally, 13 RCT studies included for this systematic review and meta-analysis.

### Study characteristics

The detailed characteristics of all included studies are described in Table 1. Nine studies used parallel design^6,14,15,18–23^ and four used cross-over design^16,17,24,25^. The intervention duration varied from 2^16,17,24^ to 48 weeks^6^. Four studies have investigated the effects of AGE diet in patients with T2DM^18–21^, six studies in Obese or overweight people^6,14–17,24^, two studies in healthy individuals^22,23^ and one study in women with PCOS^25^. Overall, ten RCTs reported changes in BMI^6,14,15,17,19,20,22–25^, seven weight^6,14–18,21^, three WC^6,14,15^ , three leptin^6,18,23^, and tree adiponectin^6,18,23^, following AGE diet consumption.

**Table 1:** Included randomized controlled trial study characteristics by population.

Study ID	Study design	Participants	Sample size	Intervention diet	Control diet	Duration	dAGE content	Outcomes
Harcourt 2011	Cross over	Overweight males aged 18–50 years and BMI 26–39 kg/m^2^)	11	LAGE diet	HAGE diet	2 weeks	HAGE = 14,090, LAGE = 3302 kU AGE/day	BMI
Uribarri 2011	Parallel	Type 2 diabetic patients	18 randomised, 18 assessed: 12 LAGE & 6 HAGE	LAGE diet	HAGE diet	4 months	HAGE = > 20, LAGE = < 10 AGE Eq/day	Weight Leptin adiponectin
de Courten 2016	Cross over	Healthy but overweight individuals	28 randomised, 20 assessed	LAGE diet	HAGE diet	2 weeks	HAGE = 59, LAGE = 49 mg CML/day	Weight
Mark 2014	Parallel	Overweight women aged 20–50 years	99 randomised, 74 assessed: 36 LAGE & 37 HAGE diet	LAGE diet	HAGE diet	4 weeks	HAGE = 24.6, LAGE = 10.7 mg CML/day	BMI Weight WC
Macías-Cervantes 2015	Parallel	Overweight men (BMI > 25 kg/m^2^) aged 30 to 55 years	75 randomised, 45 assessed: 15 in the diet plus exercise group, 14 in the exercise group, and 14 in the diet group	LAGE diet	HAGE diet	12 weeks	HAGE = 13,284 + 4983, LAGE = 13,019 + 4526 kU AGE/day	BMI Weight WC
Tantalaki 2014	Cross over	Polycystic ovary syndrome (PCOS) age range: 18–40 years	34 randomised, 23 assessed	LAGE diet	HAGE diet	8 weeks	HAGE = 1869.6 ± 114.6, LAGE = 1869.6 ± 114.6 kU AGE/day	BMI
Di Pino 2016	Parallel	Adults with prediabetes age range between 35 and 65 years; body mass index (BMI) between 18.5 and 40 kg/ m^2^	62 randomised, 57 assessed: 29 LAGE & 28 HAGE diet	LAGE diet	HAGE diet	24 weeks	N/A	BMI
Vlassara 2016	Parallel	Obese subjects with metabolic syndrome aged 50 years or above	138 randomised, 100 assessed: 51 in LAGE & 49 HAGE	LAGE diet	HAGE diet	48 weeks	HAGE = 20 + 11, LAGE = 7 + 6 AGE Eq/day	BMI Weight WC Leptin adiponectin
Cai 2004	Parallel	Diabetic patients with normal lipid profile & renal function	24 randomised: 11 HAGE & 13 LAGE	LAGE diet	HAGE diet	6 weeks	HAGE = 5 times higher than LAGE	BMI
Vlassara 2002	Parallel	Diabetic subjects with good glycemic control and normal renal function	11 for cross over and 13 for parallel trials (6 in high and 7 in low AGE diet)	LAGE diet	HAGE diet	6 weeks	HAGE = 5 times higher than LAGE	weight
Baye 2017	cross over	Overweight and obese otherwise healthy adults	204 randomised: 11 HAGE & 13 LAGE	LAGE diet	HAGE diet	2 weeks	HAGE = 3 times higher than LAGE	BMI weight
Yacoub2017	Parallel	Healthy adults aged 50–69 years	20 randomised, 20 assessed: 10 in each group	LAGE diet	HAGE diet	6 weeks	HAGE = 26.96 , LAGE = 26.18 AGE Eq/day	BMI
Uribarri 2014	Parallel	Healthy participants over the age of 60	18 randomised, 18 assessed: 10 LAGE & 6 HAGE	LAGE diet	HAGE diet	16 weeks	HAGE = > 15, LAGE = < 10 AGE Eq/day	BMI Leptin adiponectin

WC; Waist circumference; BMI; Body mass index.

### Risk of bias assessment

As shown in Table 2, six studies were assessed as unclear risk in random sequence generation, cause they did not explicitly mention the random sequence generation methods^14,18,20,21,23,24^ and one as high risk^25^. Two studies were assessed as low risk in allocation concealment^15,16^ and one as high risk^25^, while the other ten studies as unclear. There was one study which was double-blinded, thus considered as low risk of bias for blinding of participants and personnel^17^. Four of the trials provided a clear description of blinding of outcome assessment^6,15–17^. Six studies were clear on providing incomplete outcome data and then were considered as low risk of bias^6,14,16–19^. Six studies were assessed as unclear risk of bias in selective reporting^17,20–24^, and the other seven studies were assessed as low risk of bias. Except for one study that was considered as high risk in quality^25^, the other twelve studies were considered as unclear risk of bias, cause we found at least one of their six key domains of quality as unclear.

**Table 2:** Risk of bias assessment according to the Cochrane collaboration’s risk of bias assessment tool.

Study, Year (reference)	Random sequence generation	Allocation concealment	Blinding of participant and personnel	Blinding of outcome assessment	Incomplete outcome data	Selective reporting	Overall assessment of risk of bias
Harcourt 2011	Unclear	Unclear	Unclear	Unclear	Low	Low	Unclear
Uribarri 2011	Low	Unclear	Unclear	Low	Low	Low	Unclear
de Courten 2016	Unclear	Unclear	Unclear	Unclear	Unclear	Unclear	Unclear
Mark 2014	Low	Unclear	Unclear	Unclear	Low	Low	Unclear
Macías-Cervantes 2015	Low	Unclear	Low	Low	Low	Unclear	Unclear
Tantalaki 2014	Low	Low	Unclear	Low	Low	Low	Unclear
Di Pino 2016	Unclear	Unclear	Unclear	Unclear	Low	Low	Unclear
Vlassara 201(28)	Unclear	Unclear	Unclear	Unclear	Unclear	Unclear	Unclear
Cai 2004(13)	Low	Low	Unclear	Low	Unclear	Low	Unclear
Vlassara 2002	Low	Unclear	Unclear	Unclear	Unclear	Unclear	Unclear
Baye 2017	Unclear	Unclear	Unclear	Unclear	Unclear	Unclear	Unclear
Yacoub 2017	Unclear	Unclear	Unclear	Unclear	Unclear	Unclear	Unclear
Uribarri 2014	High	High	Unclear	Unclear	Unclear	Low	High

### Meta-analysis

#### Effects of low dietary AGEs on BMI

**Figure 2:**
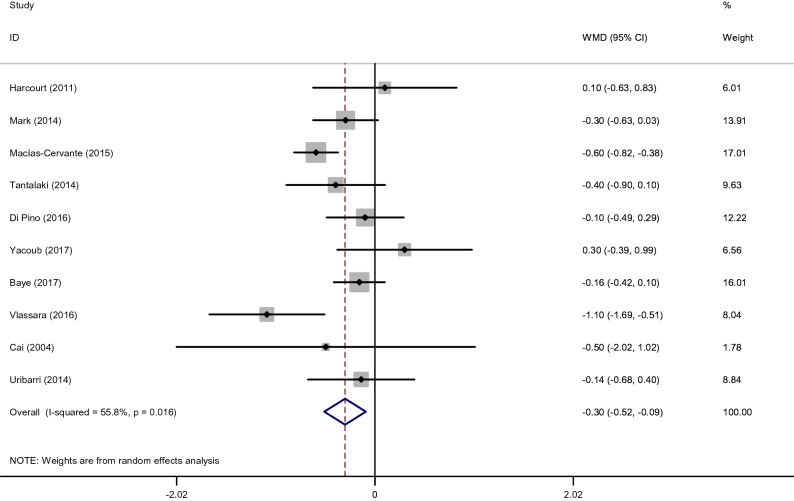
Forest plot of randomized controlled trials investigating the effects of low dietary AGEs on BMI.

The quantitative analysis of value indicated a significant reduction of BMI after consumption of the low AGE diets compared to the high AGE diets (WMD: − 0.3 kg/m^2^; 95% CI: − 0.52, − 0.09, *p* = 0.005; I^2^ = 55.8%) (Fig. 2). Our findings for the subgroup analyses showed that low AGE diets reduced BMI regardless of length of follow up. These analyses displayed that length of follow up ≥ 8 weeks (WMD: − 0.47 kg/m^2^, 95% CI: − 0.85, − 0.09) more effectively reduced BMI compared with length of follow up > 8 weeks. Also, the findings from the subgroup analyses showed that BMI levels were significantly reduced in patients with overweight or obese (WMD: − 0.41 kg/m^2^, 95% CI: − 0.72, − 0.11) (Supplemental Fig. 1**)**.

#### Effects of low dietary AGEs on Weight

**Figure 3:**
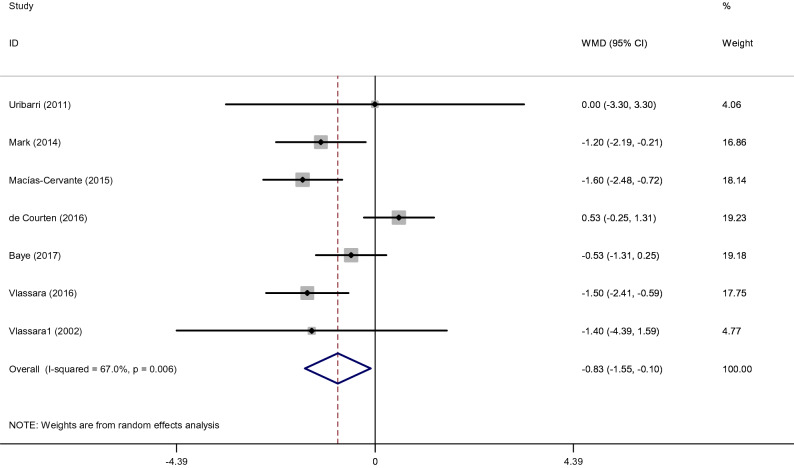
Forest plot of randomized controlled trials investigating the effects of low dietary AGEs on weight.

After intake of low AGE compared to high AGE diets, Pooled results from the random-effects model showed a significant decrease in Weight(WMD: − 0.83 kg; 95% CI: − 1.55, − 0.10, *p* = 0.026; I^2^ = 67.0%) (Fig. 3). The duration of intervention was considered as a heterogeneity factor on overall effect size. When studies were categorized based on length of follow-up, the effect of intake of low AGE compared to high AGE diets was more pronounced in subgroup with duration > 8 weeks (WMD: − 1.50 kg, 95% CI: − 2.12, − 0.88). In addition, low AGE diets were associated with a significant reduction in weight regardless of the participants’ with overweight or obese (WMD: − 0.84 kg, 95% CI: − 1.65, − 0.02) (Supplemental Fig. 2).

#### Effects of low dietary AGEs on waist circumference

**Figure 4:**
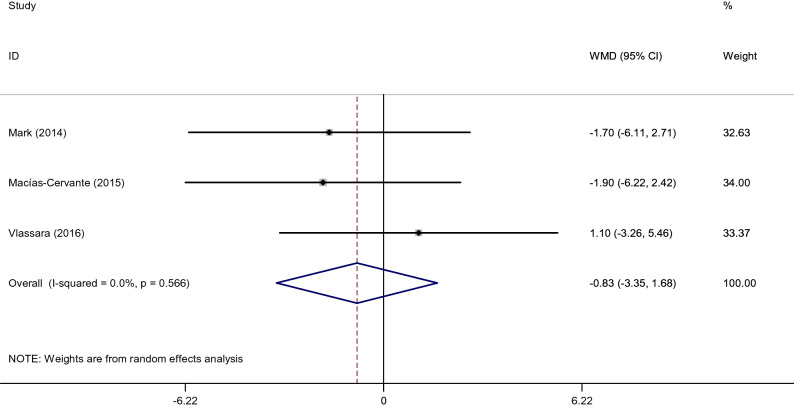
Forest plot of randomized controlled trials investigating the effects of low dietary AGEs on WC.

Among the included studies, three clinical article examined WC. After pooling effect sizes, no significant difference between low and high AGE diets with regards to WC (WMD: − 0.81 cm; 95% CI: − 2.80, 1.18, *p* = 0.43; I^2^ = 93.2%) (Fig. 4). Also, subgroup analysis for WC was not possible as there were no enough studies in each group.

#### Effects of low dietary AGEs on leptin and adiponectin

**Figure 5:**
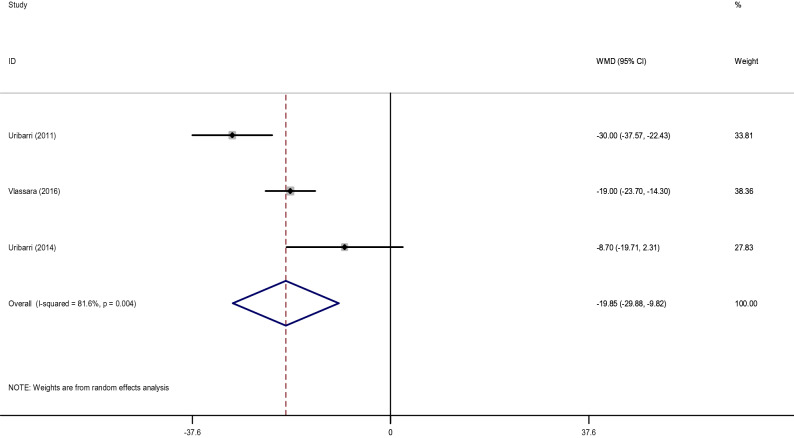
Forest plot of randomized controlled trials investigating the effects of low dietary AGEs on leptin level.

**Figure 6:**
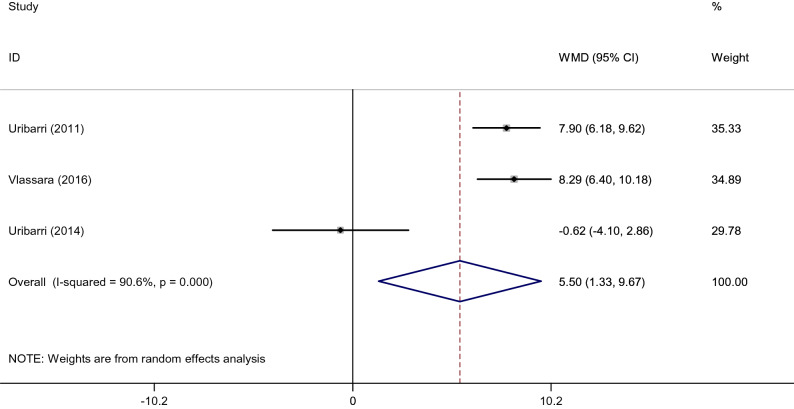
Forest plot of randomized controlled trials investigating the effects of low dietary AGEs on adiponectin level.

The pooled WMD of 3 effect sizes showed a significant decrease in leptin (WMD: − 19.85 ng/ml; 95% CI: − 29.88, − 9.82, *p* < 0.001; I^2^ = 81.8%) (Fig. 5) and an increase in adiponectin levels (WMD: 5.50 µg/ml; 95% CI: 1.33, 9.67, *p* = 0.010; I^2^ = 90.6%) (Fig. 6) after consumption of the low AGE diets compared to the high AGE diets. It was not possible to conduct subgroup analysis for leptin and adiponectin with regards to length of follow-up and health status, due to the low number of studies.

### Meta-regression results

We performed a meta‐regression analysis to examine the variation in treatment effect of low AGE diets based on duration of intervention and mean age of participants. The duration of intervention did not demonstrate any significant changes in BMI (*p* = 0.07, coef: 0.445) and weight (*p* = 0.43, coef: 0.321). Also, meta‐ regression analysis of mean age of intervention showed no significant effect on BMI (*p* = 0.86, coef: 0.630) and weight (*p* = 0.39, coef: 0.673) (supplemental Figs. 3, 4). Due to the limited number of studies on WC, leptin, and adiponectin, we could not perform the meta‐regression analysis.

### Sensitivity analysis

We removed each trial from the analysis, step by step, in order to discover the impact of each single study on the combine effect size. We observed no significant effect of any individual study on the combine effect sizes of BMI and Weight (supplemental Fig. 5). Due to the limited number of studies on WC, leptin, and adiponectin, we could not perform the analysis.

### Publication bias

A review of the funnel plots used to assess the publication bias is shown in Supplementary Fig. 6. Funnel plot showed no publication bias for BMI and Weight that were confirmed with Egger's regression test (BMI: *p* = 0.53) and (weight: *p* = 0.45).

## Discussion

This systematic review and meta-analysis was conducted to assess the effects of consumption of low dietary AGEs on obesity and its related hormone. Our findings regarding obesity indices showed that low dietary AGEs could reduce BMI and body weight but had no noticeable impact on waist circumference. Regarding obesity related hormones our results revealed a significant reduction in leptin and rise in adiponectin levels following consumption of low diet derived AGEs. These findings agree with the previous literature reporting that higher dietary consmption of AGEs was linked to increased body weight^26,27^.The evidently high amounts of AGEs in the diet especially in heat processed foods might be considered as one of the major potential factors contributting to energy over intake^26^. In a randomized controlled clinical trial, consumption of ultraprocessed foods resulted in markedly excessive energy intake and weight gain in comparision with to an unprocessed diet which caused reduction in body weight^28^. AGEs may be considered as important dietary compounds that can result in energy imbalance and subseqently increased body weight. However, the underlying mechanism by which higher AGEs content of diet may increase the risk of weight gain is not fully understood. Suggestive evidence obtained from experimental and human studies have demonstrated that higher dietary AGEs intake can induce or aggravate insulin resistance^16,29,30^. A systemaic review conduced by Clarke and colleagues demonstrated that insulin sensitivity was improved following intake of low dietary AGEs in healthy individuals and patients with type 2 diabetes mellitus. However no change in fasting glucose and HbA1c was observed^31^.

High-normal insulin levels appear to prevent lipolysis and stimulate lipogenesis in adipocytes^32^. Also an association between AGEs, insulin resistance, and weight gain has been reported in an in vivo study in Drosophila where increased methylglyoxal stimulated insulin resistance and weight gain^30^. It seems that hypothalamic inflammation is another pathway whereby higher dietary AGEs intake can increase weight. In an animal study over intake of fat and sugar stimulated hypothalamic inflammatory responses^33^. In hypothalamic inflammatory state, the signaling pathway of insulin and leptin is impaired which can lead to an adaptive increase of food consumption relative to energy expenditure that advocates weight gain^34^. However in Harcourt's study, body weight and BMI were not significantly changed after a 2 weeks either isoenergetic low or high dietary AGEs. Matching of energy intake and the short duration of the intervention might explain the null effects on BMI and body weight^24^. In case of waist circumferences, although the results of two studies^14,15^ showed beneficial effects of GAEs on WC, analysis the results of all studies^6,14,15^ showed no overall significant effects. The number of studied assessing the effects of low dietary AGEs on WC was too small. Therefore non-significant effects observed in our analysis might be related to this issue. The duration of intervention is an important factor to obtain real conclusion regarding the effects of nutritional intervention on health outcomes^7^. The length of interventions varied among studies included in this meta-analysis and the results of subgroup analysis revealed that reduction effects of low AGE diets on body weight and BMI were more pronounced in groups with length of follow up ≥ 8 weeks. Also BMI were significantly reduced in individuals with overweight or obese after intake of a low AGEs diet. Overweight and obesity are considered as the two main risk factors for the development of inflammatory process and insulin resistance^35,36^. Several Studies have demonstrated that consumption of low amount of dietary AGEs was associated with improved insulin resistance, reduced inflammatory markers and oxidative stress in overweight individuals^18,37^. Therefore the observed reduction effects of low AGEs diet on BMI might be attributed to its beneficial impacts on inflammation and insulin resistance which is more noticeable in overweight and obesity. However the results of this meta-analysis revealed that the reduction impacts of low dietary AGEs on body weigh were not significantly different between weight subgroups. This finding may be due to the variation in methodology of the studies included in subgroup analysis, differences in the type of prescribed diet or prepared meals in each study and the length of follow up. Regarding adiponectin and leptin, our finding showed that a diet with low AGEs content could significantly increase adiponectin and decrease leptin levels, two important markers of insulin resistance, suggesting that diet derived AGEs also have effects on insulin sensitive tissues^38–41^. These results are consistent with previous meta-analysis findings showing the same improvement effects of the low AGEs diet on adiponectin and leptin levels^42^. Another meta-analysis conducted by Kellow et al., has also showed that consumption of a low AGEs diet significantly decreased TNFα and 8-isoprostanes in healthy individuals^11^. SIRT1 which is a gene encoding protein belonging to the sirtuin family, is considered as a major regulator of inflammatory processes and also adiponectin levels^23^. Several studies have reported the suppression impacts of oxidant AGEs on gene expression of SIRT1^18,23^. Therefore it is thought that improving impacts of the AGE-restricted diet on insulin resistance be related to its reduction impacts on inflammatory processes, oxidative stress and leptin levels along with an increased in adiponectin and sirtuin-1^43,44^. Also, AGEs storage in adipose tissue by binding to receptors AGEs (RAGE unregulated the production of adipokines, such as adiponectin, leptin, monocyte chemotherapy protein (MCP-1), and plasminogen activator inhibitor type I (PAI-1), which Recent studies have shown that these compound poses a potential risk for cancer and other immune-related diseases through a variety of factors, such as suppressing the immune system and disrupting the regulation of monocytes, basophil, T lymphocytes, and NK cells^45,46^. AGEs-derived adipokines also appear to increase the production of reactive oxygen species and initiate anti-inflammatory signaling, which in turn further impairs the immune system^12^.

This systematic review and meta-analysis has a number of strengths. In this study, we performed a systematic review and meta-analysis on a wide range of obesity related factors including values of BMI, WC, Weight, leptin, and adiponectin that have not been reported before. Meta-analysis was also conducted according to subgroups to further detect the results of each risk factor. In addition, publication bias was checked for all of the obesity related factors. Despite the above strengths, some limitations should be considered when interpreting the results. Approximately half of the studies included in this review had poor methodological quality and length of intervention < 24 h. Therefore, we were unable to conduct a meta-analysis for these studies. In addition, differences in characteristic of prescribed diets or meals such as baseline dietary AGE levels, reliability of methods used for food preparation amongst studies must also be taken into account a confounding factor. Also, in most studies, the food was not prepared for the participants (a small number of them, but most of them only provided dietary advice on how to reduce the AGEs in their usual diet), This problem may cause a not match in the total energy received between the groups and the results may be at risk of bias.

In conclusion, our systematic reviews and meta-analyses have shown a significant decrease in BMI, weight, and leptin and an increase in adiponectin levels after consumption of the low AGE diets compared to the high AGE diets. Also, the effect of intake of low AGE compared to high AGE diets was more pronounced in subgroup with duration > 8 weeks for the BMI and weight. According to our study, low AGE diet can be effective in reducing the incidence of obesity and chronic diseases associated with high consumption of dietary AGEs. Therefore, reducing the consumption of processed foods that have high AGE content and changing food preparation methods are good strategies to promote health. Further prospective studies should be conducted in this regard.

## Methods

### Protocol

The present systematic review and meta-analysis was performed based on the principals of the PRISMA (Preferred Reporting Items for Systematic Reviews and Meta-Analyses) statement^47^.

### Search strategy

A systematic literature search was conducted in four electronic databases: Ovid MEDLINE, ISI Web of Science, Scopus, the Cochrane Central Register of Controlled Trials, and, Embase up to October, 2019. The following MeSH and text keywords were applied to identify relevant articles: (“advanced glycation end products” OR “glycation end products, advanced” OR “maillard reaction” OR “dietary advanced glycation end products” OR “circulating advanced glycation end products”) AND (obesity OR overweight OR adiponectin OR leptin) NOT review*. No language or date restrictions were imposed in the search. The detailed search strategy is provided in the Supplementary Appendix. To find relevant studies, 2 authors (Mh, S and AL) independently screened the titles, abstracts, and full texts of the retrieved articles. The references of the included reviews were also hand-searched to identify further related papers. The study protocol was previously registered with the International prospective register of systematic reviews (PROSPERO) database as **CRD42020203734.**

### Study selection

All adult clinical trials examining the impacts of low dietary intake of AGEs on obesity and related hormone were included. We excluded studies if they were (1) conducted on children; (2) assessed single food item rather than whole diet; (3) lasted < 24 h; 4) not reported sufficient data on targeted outcomes. Reviews, comments, abstracts, letters, case reports and unpublished articles were also excluded from the study.

### Data extraction

All studies were stored and managed by Endnote. After reading the selected articles all data were extracted and their integrity and reliability were assessed by two independent reviewers (Mh, S and SF) which were double checked by other authors (FSH and AL). Differences in decisions about the selected studies were resolved by consensus. Extracted information regarding each included study was as follows: title, author name(s), year of study publication, study aim, population's characteristics, sample size, study design, type of intervention (low/high AGE consumption), duration of the study duration, and, means and standard deviations of weight, BMI, WC, leptin, and adiponectin levels at baseline, post treatment and/or changes between baseline and post treatment. . Data regarding obesity related hormones and anthropometric indices were also extracted. The detailed characteristics of all included studies are described in Table 1.

### Assessment of risk of bias

The Cochrane Risk of Bias Tool for Randomized Controlled Trials^48^ was used by two authors to identify potential risks of bias. The quality assessment tool encompasses the following items: adequacy of random sequence generation, allocation concealment, blinding, the detection of incomplete outcome data as well as selective outcome reporting, and other potential sources of bias. Based on the recommendations of the Cochrane Handbook, judgment of each domain was recorded as “Low”, “High”, or “Unclear” risk of bias. Any disagreement in the data extraction and the risk of bias assessment was solved by a third reviewer.

### Data analyses

All studies were reviewed based on their main characteristic and results concerning obesity related factors. The primary outcome was Body weight, BMI and waist circumference. The serum levels of leptin and adiponectin were considered as secondary outcomes of interest.

#### Data synthesis and statistical analysis

Data were combined, if there were ≥ 3 trials within a single grouping using the generic inverse variance approach with random effects model and reported as weighted mean differences (WMDs). The random effects model and reported as weighted mean differences (WMDs) were used because included studies were performed on different populations. The statistical analysis was done using RevMan V.5.3 software and STATA version 12.0 (Stata Corp, College Station, TX, USA). If data were expressed in a different format, standard calculations were executed to obtain the mean and SDs^48,49^. For instance, if the SDs of the change were not stated in the trials, we derived it using the following formula: SD changes = square root [(SD baseline 2 + SD final 2) − (2 × R × SD baseline × SD final)]. Also, for trials that only reported standard error of the mean (SEM), SDs were obtained using the following formula: SD = SEM × √n, where “n” is the number of subjects in each group. Heterogeneity was examined using the I-squared (I^2^) statistic, in which source of heterogeneity was determined if the I^2^ value was > 50%, or if there in the case of inconsistency across RCTs data^50^. In order to identify potential sources of heterogeneity, a pre-defined subgroup analysis based on amount of low AGE, duration of intervention, and health status of subjects was performed. Meta‐regression was used to determine effect of duration of intervention and mean age of participants on outcomes. A sensitivity analysis was applied to assess the contribution of each study to the overall mean difference. We assessed the presence of publication bias using the formal Egger’s test^51^.

## Supplementary Information

Supplementary information 1.

Supplementary information 2.
