# Supplementary material for: The impact of low advanced glycation end products diet on obesity and related hormones: a systematic review and meta-analysis
Source: Sci Rep. 2020 Dec 17;10:22194. doi: 10.1038/s41598-020-79216-y (PMC7747626; doi:10.1038/s41598-020-79216-y)
Supplement: Supplementary file 1 — Supplementary information 1. [file 41598_2020_79216_MOESM1_ESM.docx]

**“The impact of low advanced glycation end products diet on obesity and related hormones; A Systematic Review and Meta-Analysis”**

Mohammad Hassan Sohouli, ^a,b^, Elham Sharifi-Zahabi ^a,b^, Abolfazl Lari ^a,b^ , Somaye Fatahi, ^a,b^**, Farzad Shidfar, ^a^ *,

^a^ Department of Nutrition, School of Public Health, Iran University of Medical Sciences, Tehran, Iran

^b^ Student Research Committee, Faculty of public health Branch, Iran University of medical sciences, Tehran, Iran

***First corresponding author:**

Farzad Shidfar

Address:

Department of Nutrition, School of Public Health, Iran University of Medical Sciences, Hemmat superhighway, Tehran, Iran.

Phone: +098 21 88602218

Fax: +098 21 88602219

Email : farzadshidfar@yahoo.com

****co-first Author:**

Somaye Fatahi

a)

b)

**Supplementary figure1.** Forest plot of randomized controlled trials investigating the effects of low dietary AGEs on BMI based on 1) type of population and b) duration.

a)

b)

**Supplementary figure2.** Forest plot of randomized controlled trials investigating the effects of low dietary AGEs on weight based on 1) type of population and b) duration.

a) b)

**Supplementary figure 3.** meta‐regression analysis to examine the variation in treatment effect of low AGE diets based on a) duration of intervention [weeks] and b) mean age of participants [years] for BMI.

a) b)

**Supplementary figure 4.** meta‐regression analysis to examine the variation in treatment effect of low AGE diets based on a) duration of intervention [weeks] and b) mean age of participants [years] for weight.

a) b)

**Supplementary figures 5.** Sensitivity analysis of the weighted mean difference (WMD) versus the standard error (s.e) for a) BMI and b) weight.

a) b)

**Supplementary figures 6.** Funnel plot of the weighted mean difference (WMD) versus the standard error (s.e) for a) BMI and b) weight.
