# Supplementary material for: The impact of low advanced glycation end products diet on obesity and related hormones: a systematic review and meta-analysis
Source: Sci Rep. 2020 Dec 17;10:22194. doi: 10.1038/s41598-020-79216-y (PMC7747626; doi:10.1038/s41598-020-79216-y)
Supplement: Supplementary file 2 — Supplementary information 2. [file 41598_2020_79216_MOESM2_ESM.docx]

| ***Supplementary Appendix Text.***  ***Ovid MEDLINE*** *((Diet[Mesh]) OR diet[Title/Abstract]) OR food[Mesh]) OR food[Title/Abstract]) OR eating[Mesh]) OR eating[Title/Abstract]) OR “nutritive value”[Title/Abstract]) OR “nutrtion therapy”[Title/Abstract]) OR “diet therapy”[Title/Abstract]) OR meals[Mesh]) OR meals[Title/Abstract]) OR “energy intake”[Title/Abstract]) OR food*[Title/Abstract]) OR diet*[Title/Abstract]) OR "food restrict*"[Title/Abstract]) OR "food regimen*"[Title/Abstract]) OR nutrtion[Title/Abstract]) OR “calorie intake”[Title/Abstract]) OR "nutrient* intake"[Title/Abstract]) OR “food modif*”[Title/Abstract]) OR “meal modif*”[Title/Abstract] OR “meal restrict*”[Title/Abstract]) OR “diet* regimen*”[Title/Abstract]) OR “diet* program*”[Title/Abstract]) OR “diet* therap*”[Title/Abstract]) OR “nutrtion therap”[Title/Abstract])*  1. *((Glycosylation End Products, Advanced[Mesh]) OR “Glycosylation End Products, Advanced”[Title/Abstract]) OR glycosylation[Mesh]) OR glycosylation[Title/Abstract]) OR “maillard reaction”[Mesh]) OR “maillard reaction”[Title/Abstract]) OR “advanced glyc*”[Title/Abstract]) OR maillard [Title/Abstract]) OR "browning reaction"[Title/Abstract]) OR "high oxidant compounds"[Title/Abstract]) OR "nepsilon carboxymethyllysine"[Title/Abstract]) OR "methyl glyoxal"[Title/Abstract]) OR “advanced lipox*”[Title/Abstract]) OR “glycat* stress”[Title/Abstract]) OR "soluble receptor of advanced glycation end products"[Title/Abstract]) OR sRAGE[Title/Abstract]) OR "endogenous secretory receptor for advanced glycation end products"[Title/Abstract]) OR esRAGE[Title/Abstract]) OR glycation[Title/Abstract]) OR thermal[Title/Abstract]* 2. *((randomized controlled trial[Publication Type]) OR “controlled clinical trial”[Publication Type]) OR intervention[Title/Abstract]) OR intervention*[Title/Abstract]) OR trial[Title/Abstract]) OR randomized[Title/Abstract]) OR randomised[Title/Abstract]) OR random[Title/Abstract]) OR randomly[Title/Abstract]) OR placebo[Title/Abstract]) OR assignment[Title/Abstract]) OR RCT[Title/Abstract]) OR clinical trial[MeSH Terms]*   ***Scopus***   1. *TITLE-ABS-KEY ( diet* )  OR  TITLE-ABS-KEY ( food* )  OR  TITLE-ABS-KEY ( food  W/2  restrict* )  OR  TITLE-ABS-KEY ( food  AND regimen* )  OR  TITLE-ABS-KEY ( nutrition )  OR  TITLE-ABS-KEY ( nutrition*  AND therap* )  OR  TITLE-ABS-KEY ( diet*  AND regim* )  OR  TITLE-ABS-KEY ( diet*  AND program* )  OR  TITLE-ABS-KEY ( diet*  AND therap* )  OR  TITLE-ABS-KEY ( meal* )  OR  TITLE-ABS-KEY ( meal  W/2  restrict* )  OR  TITLE-ABS-KEY ( energy  AND intake )  OR  TITLE-ABS-KEY ( calorie  AND intake )  OR  TITLE-ABS-KEY ( food  W/2  modif* )  OR  TITLE-ABS-KEY ( meal  W/2  modif* )  OR  TITLE-ABS-KEY ( nutrient*  W/2  intake )*  *2- TITLE-ABS-KEY (*advanced*AND*glyc**)  OR  TITLE-ABS-KEY (*maillard*)  OR  TITLE-ABS-KEY (*browning*W/2* reaction*)  OR  TITLE-ABS-KEY (*high*AND*oxidant*AND*compounds*)  OR  TITLE-ABS-KEY (*nepsilon*W/2* carboxymethyllysine*)  OR  TITLE-ABS-KEY (*methyl*W/2* glyoxal*)  OR  TITLE-ABS-KEY (*advanced*AND*lipox**)  OR  TITLE-ABS-KEY (*glycat**AND*stress*)  OR  TITLE-ABS-KEY (*soluble*AND*receptor*AND*of*AND*advanced*AND*glycation*AND*end*AND*products*)  OR  TITLE-ABS-KEY (*srage*)  OR  TITLE-ABS-KEY (*endogenous*AND*secretory*AND*receptor*AND*for*AND*advanced*AND*glycation*AND*end*AND*products*)  OR  TITLE-ABS-KEY (*esrage*)  OR  TITLE-ABS-KEY (*glycation*)  OR  TITLE-ABS-KEY (*thermal*)**3- ( TITLE-ABS-KEY ( clinical  AND trial* )  OR  TITLE-ABS-KEY ( controlled  AND trial* )  OR  TITLE-ABS-KEY ( random* )  OR  TITLE-ABS-KEY ( placebo* )  OR  TITLE-ABS-KEY ( "double blind*" )  OR  TITLE-ABS-KEY ( "single blind*" )  OR  TITLE-ABS-KEY ( intervention* )  OR  TITLE-ABS-KEY ( rct ) )* ***Embase***   - 1. *'glycosylation' OR 'glycosylation'/exp OR glycosylation OR 'advanced glycation end product'/exp OR 'advanced glycation end product' OR 'advanced glycation end product receptor'/exp OR 'advanced glycation end product receptor' OR 'advanced glyc*' OR 'maillard reaction product'/exp OR 'maillard reaction product' OR (browning NEXT/2 reaction) OR 'glycation'/exp OR 'glycation' OR 'advanced lipox*' OR (nepsilon NEXT/2 carboxymethyllysine) OR (methyl NEXT/2 glyoxal) OR srage OR 'endogenous secretory receptor for advanced glycation end products' OR esrage OR thermal OR glycat* OR 'soluble receptor of advanced glycation end products'*   2. *((random* OR factorial* OR crossover*) AND (orcross NEXT/1 over*) OR placebo* OR doubl*) AND blind* OR (singl* NEXT/2 blind*) OR assign* OR allocat* OR volunteer* OR 'cross over procedure'/exp OR 'cross over procedure' OR 'double blind procedure'/exp OR 'double blind procedure' OR 'randomized controlled trial'/exp OR 'randomized controlled trial' OR 'single blind procedure'/exp OR 'single blind procedur'*   3. *diet* OR food* OR (food NEXT/2 restrict*) OR 'food regimen*' OR nutrition OR 'nutrition* therap*' OR 'diet* regim*' OR 'diet* program*' OR 'diet* therap*' OR meal* OR (meal NEXT/2 restrict*) OR 'energy intake' OR 'calorie intake' OR (food NEXT/2 modif*) OR (meal NEXT/2 modif*) OR (nutrient* NEXT/2 intake)*   ***web of scince***  *1. ( TS= (glycosylation) OR TS=("advanced glycation end product") OR TS=("advanced glycation end product receptor") OR TS=("advanced glyc*") OR TS= ("maillard reaction product") OR TS=("maillard reaction product") OR TS=("browning reaction") OR TS=(glycation) OR TS=("advanced lipox*") OR TS= ("nepsilon carboxymethyllysine") OR TS=("methyl glyoxal") OR TS=(srage) OR TS=("endogenous secretory receptor for advanced glycation end products") OR TS=(esrage) OR TS=(thermal) OR TS=("soluble receptor of advanced glycation end products") OR TS=(glycat*))*  *2. ( TS= (diet*) OR TS=(food) OR TS=("food regim*") OR TS=("food restrict*") OR TS= ("nutrtion therap*") OR TS=("diet* restrict*") OR TS=("diet* regim*") OR TS=(nutrient*) OR TS=("diet* program*") OR TS= ("diet* therap*") OR TS=("meal restrict*") OR TS=(meal) OR TS=("energy intake") OR TS=(“calorie intake”) OR TS=(“meal modif*”) OR TS=("food modif*") OR TS=(“nutrient* intake”))*  *3. (TS=( clinical AND trial* ) OR TS=( controlled AND trial* ) OR TS=( random* ) OR TS= ( placebo* ) OR TS=( "double blind*" ) OR TS=( "single blind*" ) OR TS=( intervention* ) OR TS=( rct ) )*  ***Cochraine***  *glycosylation OR "advanced glycation end product" OR "advanced glycation end product receptor" OR "advanced glyc*" OR "maillard reaction product" OR "maillard reaction product" OR "browning reaction" OR glycation OR "advanced lipox*" OR "nepsilon carboxymethyllysine" OR "methyl glyoxal" OR srage OR "endogenous secretory receptor for advanced glycation end products" OR esrage OR thermal OR "soluble receptor of advanced glycation end products" OR glycat** |
| --- |
